# Comparative Profiling of Antibiotic Resistance Genes and Microbial Communities in Pig and Cow Dung from Rural China: Insights into Environmental Dissemination and Public Health Risks

**DOI:** 10.3390/biology14111623

**Published:** 2025-11-19

**Authors:** Haifeng Wang, Juan Guo, Xing Chen

**Affiliations:** Kaifeng Key Laboratory of Food Composition and Quality Assessment, School of Environmental Engineering, Yellow River Conservancy Technical University, Kaifeng 475004, China

**Keywords:** antibiotic resistance genes (ARGs), environmental contamination, microbial communities, mobile genetic elements (MGEs), pig and cow dung, public health risk

## Abstract

Antibiotic resistance genes (ARGs) and microbial communities in pig and cow dung from rural China were systematically profiled using high-throughput quantitative PCR arrays and 16S rDNA amplicon sequencing to assess their environmental dissemination and public health risks. The abundance and diversity of ARGs were markedly higher in pig dung than in cow dung. A total of 56 ARGs were enriched in pig dung, including β-lactamase genes (*blaCMY*, *blaCTX-M*) and macrolide resistance genes (*ermB*, *ermF*), along with several genes related to aminoglycoside and macrolide–lincosamide–streptogramin B resistance. In contrast, only eight ARGs were enriched in cow dung. Microbial community analysis revealed that cow dung was dominated by *UCG-005*, *UCG-010*, *Methanocorpusculum*, and *Fibrobacter*, taxa typically associated with ruminant digestion. In pig dung, *Ignatzschineria*, *Lactobacillus*, *Pseudomonas*, *Streptococcus*, *Treponema*, and conditional pathogens such as *Escherichia coli* and *Leptospira* were significantly enriched, indicating higher pathogen-related risks. Functional prediction identified 26 KEGG level-2 and 136 level-3 pathways, showing stronger xenobiotic degradation and amino acid metabolism in pig dung, whereas cow dung was enriched in energy metabolism and chemotaxis pathways. Moreover, the higher abundance of mobile genetic elements (e.g., *intI1* and *IS613*) in pig dung suggests a greater potential for horizontal ARG transfer. Integrating ARG, microbial, and pathogen data reveals that pig dung acts as a composite source of “ARG–pathogen” contamination with enhanced transmission potential. These findings provide localized, data-driven evidence for developing safer livestock waste management practices, such as composting and biogas utilization, and contribute to antibiotic resistance mitigation strategies in rural China.

## Simple Summary

Livestock dung is an important source of nutrients for agriculture but can also act as a reservoir for antibiotic resistance genes (ARGs) and harmful microorganisms that threaten environmental and public health. In this study, we compared pig and cow dung collected from small farms in rural China using high-throughput PCR arrays and 16S rDNA sequencing. We found that pig dung contained far more ARGs and potential pathogens than cow dung. Genes related to β-lactam and macrolide resistance were particularly abundant in pig dung, which also showed higher levels of mobile genetic elements that can promote ARG transfer. Cow dung, by contrast, was dominated by microorganisms associated with digestion in ruminants. These findings highlight that pig dung represents a higher risk of spreading antibiotic resistance and pathogens to the environment. Our results provide useful information for developing safer dung management practices, such as composting and biogas treatment, to reduce antibiotic resistance risks in rural livestock systems.

## 1. Introduction

Excessive use of antibiotics in medicine, agriculture, and livestock production has led to a global increase in antibiotic resistance driven by microorganisms carrying multiple antibiotic resistance genes (ARGs) [1]. Increasing antibiotic concentrations in the environment exert a selective pressure that favors the growth of antibiotic-resistant bacterial strains [2], posing substantial threats to human health [3]. The presence of ARGs and microbial communities in livestock dung poses significant environmental and public health risks, particularly in regions with intensive animal husbandry [4,5,6].

In rural China, small-scale cow and pig farming remains widespread and is often characterized by non-standard feeding practices and inadequate dung management, which may exacerbate the dissemination of ARGs and pathogenic microorganisms. The overuse of antibiotics in livestock production is a key driver of ARG proliferation [7]. In the gut microbiota of livestock and poultry, ARGs can spread to soil, water, and crops through dung application, runoff, and other pathways, forming a “breeding–environment–human” resistance transmission chain [8]. For example, when untreated livestock dung is applied to fields or used in biogas systems, ARGs can be transferred to environmental microorganisms via horizontal gene transfer and may even reach the human gut through the food chain, potentially leading to antibiotic treatment failure [9,10].

As one of the world’s largest livestock-producing countries, China’s rural areas predominantly rely on small-scale farming, where common challenges include non-standard feed use, antibiotic overuse, and a lack of safe dung treatment [11,12]. To reduce costs, rural farmers often use inexpensive feed containing antibiotics but lack facilities for safe dung disposal, resulting in the accumulation of ARGs and pathogenic microorganisms. The dung microbiota of cows and pigs also differ due to their distinct digestive systems (ruminant vs. monogastric). The bovine gut is dominated by fiber-degrading and methanogenic bacteria, whereas the porcine gut tends to harbor conditional pathogens such as *Proteobacteria* [13,14]. These differences may contribute to variation in the distribution and transmission risk of ARGs.

However, systematic comparative studies on ARG spectra, microbial composition, and pathogen-associated risks in cow and pig dung from small-scale rural farms remain scarce. Such research is essential for understanding how dung-associated microbial communities influence ARG dissemination and environmental health [15]. The high ARG abundance in pig dung may also reflect farming practices that shape the resistome and contribute to human exposure through environmental pathways [16]. Moreover, in rural areas, livestock dung is often mixed with agricultural waste and domestic sewage, forming complex microbial ecosystems that facilitate ARG and mobile genetic element (MGE) transmission [13,17,18]. The co-occurrence of ARGs and opportunistic pathogens in dung increases the risk of zoonotic disease and environmental contamination [19,20,21]. Despite growing interest, the synergistic distribution and composite risks of ARGs and pathogenic microorganisms—such as *Prevotella* spp., *Campylobacter fetus*, *Orfvirus*, *Lactobacillus crispatus*, and *Lawsonia intracellularis*—remain poorly understood [22,23,24].

This study investigated cow and pig dung collected from smallholder farms in Henan Province, China. By combining high-throughput quantitative PCR and 16S rDNA amplicon sequencing, we systematically compared ARG profiles, microbial community structures, and metabolic functions between cow and pig dung, aiming to provide localized scientific evidence for mitigating antibiotic resistance and improving dung safety in rural agricultural systems.

## 2. Materials and Methods

### 2.1. Sample Collection

Fresh cow and pig dung samples were collected in the spring of 2024 from two different smallholder farms located in different rural townships of Kaifeng, Henan Province, China. Sampling during the same season minimized potential seasonal fluctuations in microbial composition. Both farms represent typical non-standardized, small-scale livestock operations that are widespread in central China.

The livestock houses at both sites were brick-built and naturally ventilated, and neither farm implemented formal sanitation or disinfection programs. Feeding practices were generally similar across the two farms: animals were mainly provided commercial feed supplemented with diverse household- or farm-sourced auxiliary materials, such as crop residues or other mixed-origin feed components. However, the farmers did not keep detailed records of feed ingredients and types, animal health status, or antibiotic use, and these uncontrolled factors may influence microbial communities and antibiotic resistance gene (ARG) profiles.

Dung was typically accumulated in uncovered farmyard areas without treatment, increasing the potential for environmental contamination and human exposure to harmful microorganisms. These management characteristics highlight the importance of studying microbial composition and antibiotic resistance under non-standardized smallholder livestock systems, which remain insufficiently documented in this region. 

For each livestock species, five independent dung samples were collected for 16S rDNA amplicon sequencing, and three samples were collected for quantitative PCR (qPCR) array analysis. Samples from each species were taken from spatially separated fresh dung piles within the same farm, ensuring sample independence while reflecting the natural variability present under real-world farming conditions.

### 2.2. Isolation and Extraction of Bacterial Genomic DNA

Genomic DNA was extracted from cow and pig dung samples using a SPINeasy DNA Kit (MP Biomedicals, Santa Ana, CA, USA) according to the manufacturer’s protocol. The quality of extracted DNA was verified using 1% agarose gel electrophoresis, and the concentration and purity were measured with a NanoDrop One spectrophotometer (Thermo Fisher Scientific, Waltham, MA, USA). DNA samples were stored at −20 °C until further use.

### 2.3. PCR Array Analysis

The commercial PCR Array Kit (wc-DNABAL230517, WcGene Biotech, Shanghai, China) contained a mixed set of primers targeting bacteria-specific gene regions as well as selected viral and parasitic marker genes. This design allowed simultaneous detection of multiple microbial categories, including bacteria, viruses, and parasites. The types of ARGs and specific primer sequences used for ARG detection are listed in Table S1, and the microorganisms and their primer sequences are listed in Table S2. Quantitative PCR was performed using a QuantStudio 5 Real-Time PCR System (Applied Biosystems, Foster City, CA, USA). Each 10 μL reaction contained 5 μL iTaq Universal SYBR Green Supermix (Bio-Rad, Hercules, CA, USA), 1 μL DNA template, 1 μL of forward and reverse primers, and 3 μL double-distilled water. The thermal cycling program consisted of an initial denaturation at 98 °C for 3 min, followed by 40 cycles of 98 °C for 15 s and 60 °C for 30 s. A melting curve analysis (60–95 °C) was performed to verify amplification specificity. Gene abundance levels were normalized to 16S rDNA, and relative abundance was calculated using the comparative CT method (2^−ΔΔCT^). The average CT values were obtained as follows: ΔCT = CT(target gene) − CT(16S rDNA), and ΔΔCT = ΔCT(sample) − ΔCT(reference).

### 2.4. 16S rDNA Sequencing Analysis

The total DNA quality was verified using a NanoDrop 2000 spectrophotometer (Thermo Fisher Scientific) and 1% agarose gel electrophoresis. The 16S rDNA V3–V4 regions were amplified using the universal primers 341F (5′-CCTACGGGRSGCAGCAG-3′) and 806R (5′-GGACTACVVGGGTATCTAATC-3′). PCR amplification was carried out in a 25 μL reaction containing 12.5 μL of KFX HiFi 2× PCR Master Mix, (ABclonal, Wuhan, China) 1 μL of each primer (10 μM), 50 ng of template DNA, and ddH_2_O. Negative controls consisting of sterile tubes were processed alongside samples, yielding no detectable amplification. PCR products were separated on 2% agarose gels and purified using an AxyPrep DNA Gel Extraction Kit (Axygen Biosciences, Union City, CA, USA). Amplicons were quantified using a Qubit^®^ 2.0 fluorometer (Invitrogen, Carlsbad, CA, USA) and pooled in equimolar ratios for sequencing on an Illumina NovaSeq platform (San Diego, CA, USA). Primer sequences were trimmed using Cutadapt in QIIME2 (v2022.2). DNA extraction, library construction, and sequencing were performed by RealBio Genomics Institute (Shanghai, China).

### 2.5. Process of Sequencing Data

The DADA2 plug-in in QIIME2 (v2022.2) was used to filter, denoise, and merge non-chimeric reads after primer removal, generating amplicon sequence variants (ASVs). Representative sequences were assigned taxonomic classifications based on the SILVA database (v138).

### 2.6. Statistical Analysis

Independent-samples *t*-tests were used for normally distributed variables, with *p* < 0.05 considered statistically significant. Analysis of similarities (ANOSIM) was performed using the vegan package in R (3.5.1). Principal coordinate analysis (PCoA) was conducted using the ade4 package in R (3.5.1). Linear discriminant analysis (LDA) effect size (LEfSe) was applied to identify bacterial taxa differing in abundance between groups. Metabolic pathway analysis of bacterial communities was conducted using PICRUSt, and LEfSe was further applied to estimate the contribution of each pathway to observed group differences.

## 3. Results

### 3.1. Analysis of Microbial ARGs in Cow and Pig Dung Samples

**Figure 1:**
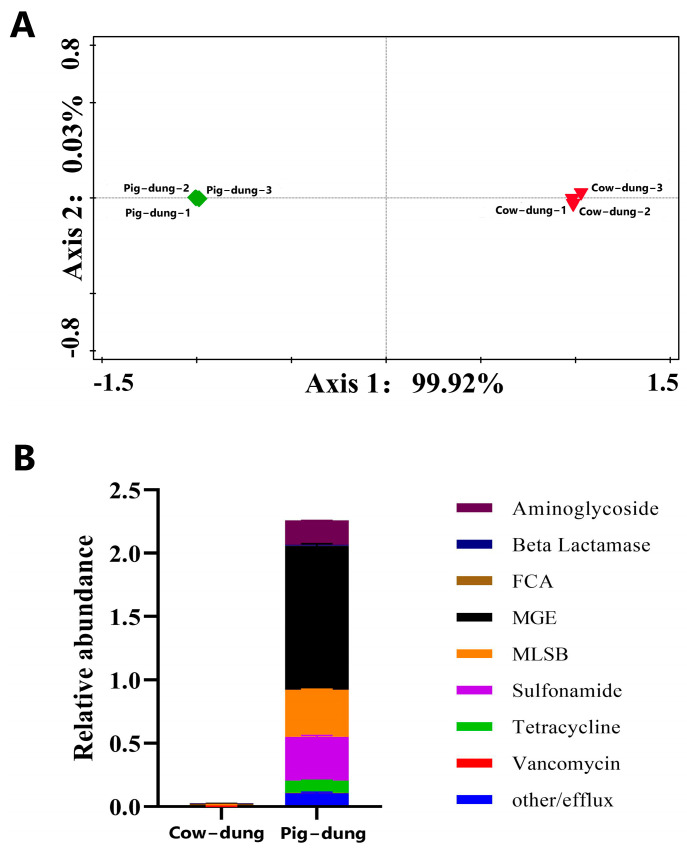
Fluorescence quantitative detection results of ARG profiles in cow dung and pig dung. (**A**) PCoA results of cow and pig dung samples. (**B**) Stacked histograms depicting the ARG abundance in cow and pig dung samples. Different colors indicate various antibiotic types, with ARG abundance quantified as the number of ARG copies per copy of the 16S rDNA gene. ARG: antibiotic resistance gene; PCoA: principal coordinate analysis.

Based on the expression of ARGs, PCoA distinguished between cow and pig Dung samples, with each group clustered together (Figure 1A). The results of PCoA demonstrated significant differences in the ARG profiles between the two groups, with minor variations within each group.

To determine the differences in aminoglycosides; beta-lactamase; fluoroquinolone, quinolone, florfenicol, chloramphenicol, and amphenicol resistance; MGEs; macrolide-lincosamide-streptogramin B (MLSB); sulfonamide; tetracycline; vancomycin; and other/efflux ARGs, we conducted high-throughput qPCR and expression analysis based on the gene expression abundance (Figure 1B). The results showed that the expression of the above nine categories of ARGs was higher in pig Dung samples than in cow dung samples.

**Figure 2:**
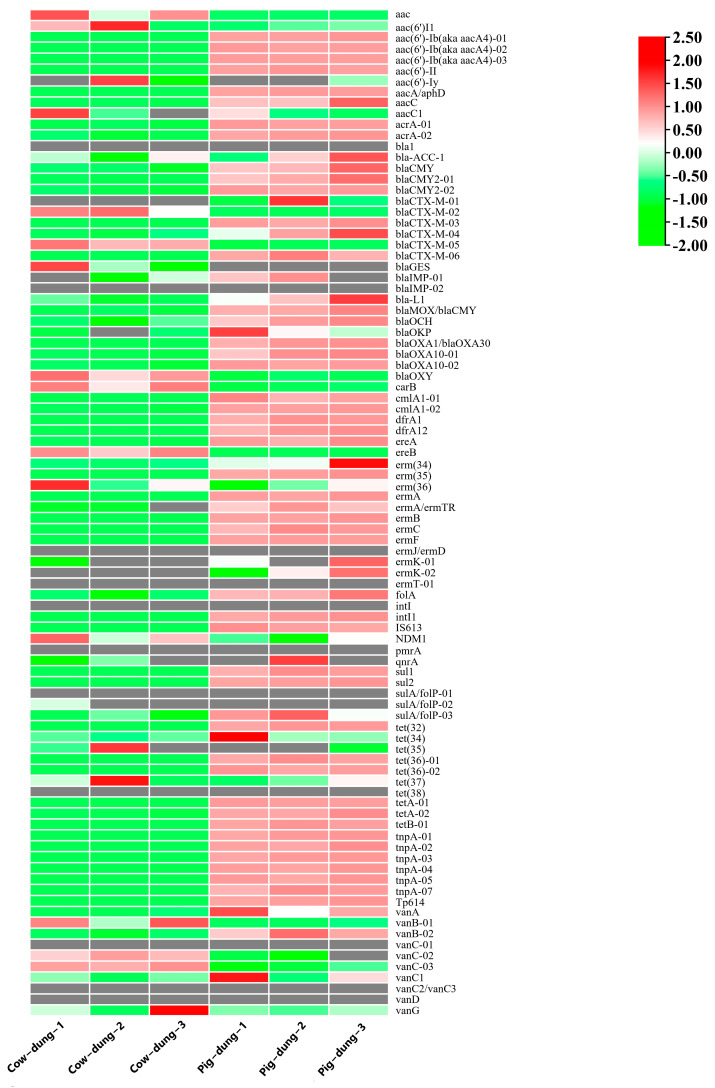
Expression heatmaps of ARGs in different cow and pig dung samples. Red and green indicate up- and downregulation, respectively.

Analysis of 92 specific ARGs indicated prevalent upregulation of ARGs in pig Dung samples, including *aac(6′)-Ib* (also known as *aacA4*)*-01*, *aac(6′)-Ib* (also known as *aacA4*)-*02*, *aac(6′)-Ib* (also known as *aacA4*)-*03*, *aacA*/*aphD*, *aacC*, *acrA-01*, *acrA-02*, *blaCMY*, *blaCMY2-01*, *blaCMY2-02*, *blaCTX-M-03*, *blaCTX-M-04*, *blaCTX-M-06*, *bla-L1*, *blaMOX*/*blaCMY*, *blaOCH*, *blaOXA1*/*blaOXA30*, *blaOXA10-01*, *blaOXA10-02*, *cmlA1-01*, *cmlA1-02*, *dfrA1*, *dfrA12*, *ereA*, *erm*(35), *ermA*, *ermA*/*ermTR*, *ermB*, *ermC*, *ermF*, *folA*, *intI1*, *IS613*, *sul1*, *sul2*, *tet(32)*, *tet (36)-01*, *tet(36)-02*, *tetA-01*, *tetA-02*, *tetB-01*, *tnpA-01*, *tnpA-02*, *tnpA-03*, *tnpA-04*, *tnpA-05*, *tnpA-07*, *Tp614*, and *vanB-02*. An expression heatmap of ARGs in the cow and pig Dung samples is shown in Figure 2. These findings demonstrate the substantial impact of ARG dynamics on cow and pig Dung.

### 3.2. Differentially Expressed ARGs Between Cow and Pig Dung Samples

**Figure 3:**
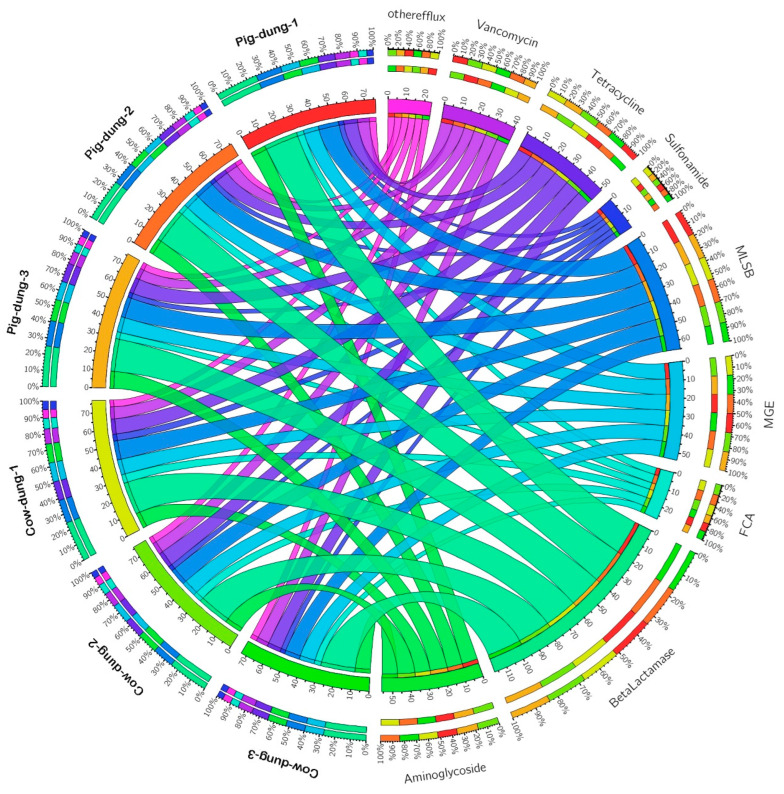
Number and percentage of differentially expressed ARGs in each cow or pig dung sample.

To explore the differentially expressed ARGs between cow and pig dung samples, we analyzed the fold-change (FC) and *p* values of these genes. Using the criteria of log_2_FC > 1 and *p* < 0.05, we identified 58 differentially expressed ARGs between cow and pig dung samples. Compared with cow dung samples, eight ARGs including *aac*, *blaCTX-M-02*, *blaCTX-M-05*, *carB*, *ereB*, *vanB-01*, *vanC-02*, and *vanC-03* were downregulated in pig dung samples, whereas 48 ARGs, including *aac(6′)-Ib* (also known as *aacA4*)-*01*, *aac(6′)-Ib* (also known as *aacA4*)-*02*, *aac(6′)-Ib* (also known as *aacA4*)-*03*, *aac(6′)-II*, *aacA*/*aphD*, *aacC*, *acrA-01*, *acrA-02*, *blaCMY*, *blaCMY2-01*, *blaCMY2-02*, *blaCTX-M-03*, *blaCTX-M-04*, *blaCTX-M-06*, *blaMOX*/*blaCMY*, *blaOCH*, *blaOXA1*/*blaOXA30*, *blaOXA10-01*, *blaOXA10-02*, *cmlA1-01*, *cmlA1-02*, *dfrA1*, *dfrA12*, *ereA*, *erm(35)*, *ermA*, *ermA*/*ermTR*, *ermB*, *ermC*, *ermF*, *folA*, *intI1*, *IS613*, *sul1*, *sul2*, *tet(32)*, *tet(36)-01*, *tet(36)-02*, *tetA-01*, *tetA-02*, *tetB-01*, *tnpA-01*, *tnpA-02*, *tnpA-03*, *tnpA-04*, *tnpA-05*, *tnpA-07*, *Tp614*, *vanA*, and *vanB-02* were upregulated (Figure S1). To determine the distribution of each type of ARG across different samples, circular graphs were used to depict both the number and percentage of ARGs. ARGs associated with aminoglycosides; beta-lactamase; fluoroquinolone, quinolone, florfenicol, chloramphenicol, and amphenicol; MGEs; MLSB; sulfonamide; tetracycline; vancomycin; and other/efflux were present in all samples. Notably, beta-lactamase, MLSB, aminoglycoside, and tetracycline resistance genes showed the largest numbers and proportions in both cow and pig dung (Figure 3). These data suggest that ARGs accumulate and persist in pig dung.

### 3.3. Analysis of 16S rDNA Sequencing Data

After 16S rDNA amplicon sequencing, a total of 471,031 raw reads (cow dung = 245,002; pig dung = 226,029) and 443,073 filtered reads (cow dung = 229,600; pig dung = 213,473) were obtained. The effective sequence ratios were 93.71% and 94.44% for cow and pig dung samples, respectively (Table S3). Principal component analysis (PCA) based on amplicon sequence variants (ASVs) showed a clear separation between cow and pig dung samples, confirming data reliability and adequacy for further analysis (Figure S2A). In the Venn diagram, different colors represent distinct ASV groups: cow dung contained 727 ASVs, pig dung contained 582 ASVs, and eight ASVs were shared between the two groups (Figure S2B).

### 3.4. Comparative Evaluation of Dung Microbiota Variations Between Two Groups

**Figure 4:**
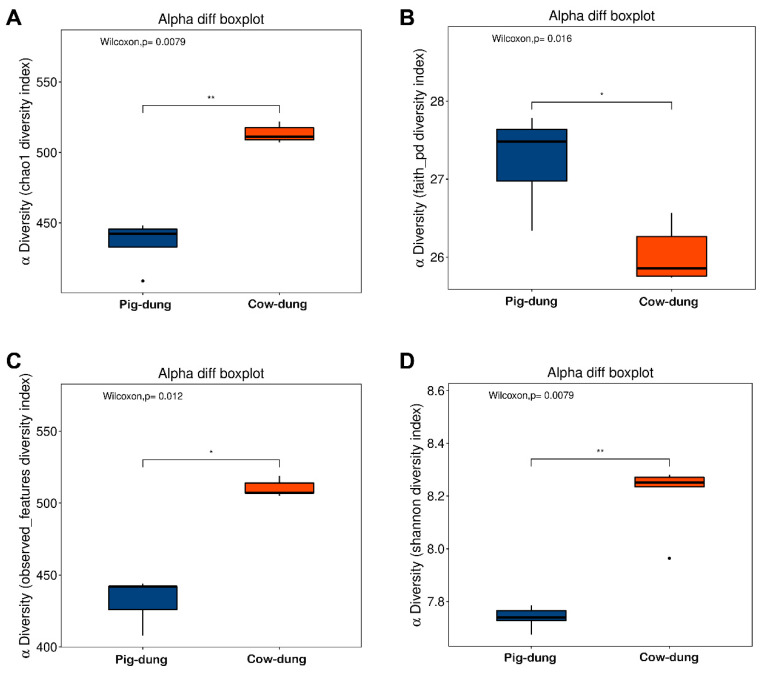
Alpha diversity in dung bacteria between pig and cow dung samples based on four indices: Chao1 (**A**), Faith_pd (**B**), Observed_features (**C**), and Shannon index (**D**). * *p* < 0.05 and ** *p* < 0.01.

The alpha diversity of the dung microbial fraction of cow and pig dung samples was evaluated using four indices: Chao1 represented species richness (Figure 4A), Faith_pd represents phylogenetic diversity (Figure 4B), Observed_features represents direct counts of observed species (Figure 4C), and the Shannon index represents the overall biodiversity based on both species’ richness and evenness (Figure 4D). Compared with cow dung samples, Chao1, Observed_features, and Shannon indices were lower in pig dung samples, whereas Faith_pd was higher, indicating lower microbial diversity in pig dung samples than in cow dung samples. Beta diversity analysis based on PCoA showed that the samples were distantly and significantly separated between groups (Figure S3A). Non-metric multidimensional scaling revealed a significant difference between cow and pig dung samples, with mean values of 0.36 and −0.36, respectively; the standard deviation of each was 0, indicating high stability within the same sample type. There were long distances between samples of cow and pig dung groups, indicating that the dung bacterial community was species-specific (Figure S3B). Analysis of similarities indicated that intergroup differences were significantly greater than within-group differences (*p* = 0.01; Figure S3C). Using cluster analysis, the distances between samples were calculated to determine the similarity in species composition. The unweighted UniFrac heatmap for all samples is shown in Figure S3D, where closer samples indicate a more similar species composition.

### 3.5. Important Bacteria in Cow and Pig Dung Samples

**Figure 5:**
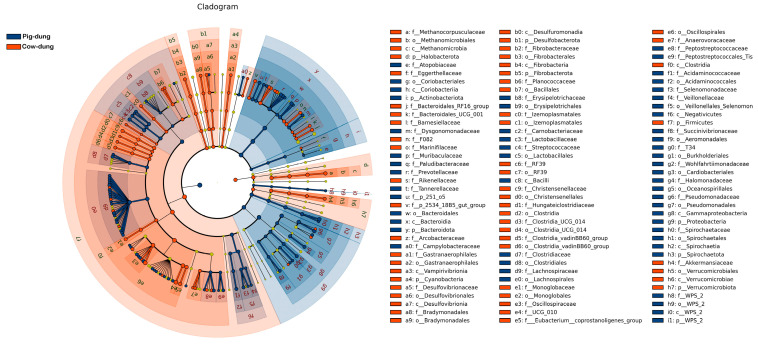
Cladogram showing differential bacterial taxa identified by LEfSe analysis between cow and pig dung samples. Different colors represent distinct groups, and nodes of corresponding colors indicate microbial taxa that play key roles in each group. LEfSe, Linear Discriminant Analysis Effect Size.

**Figure 6:**
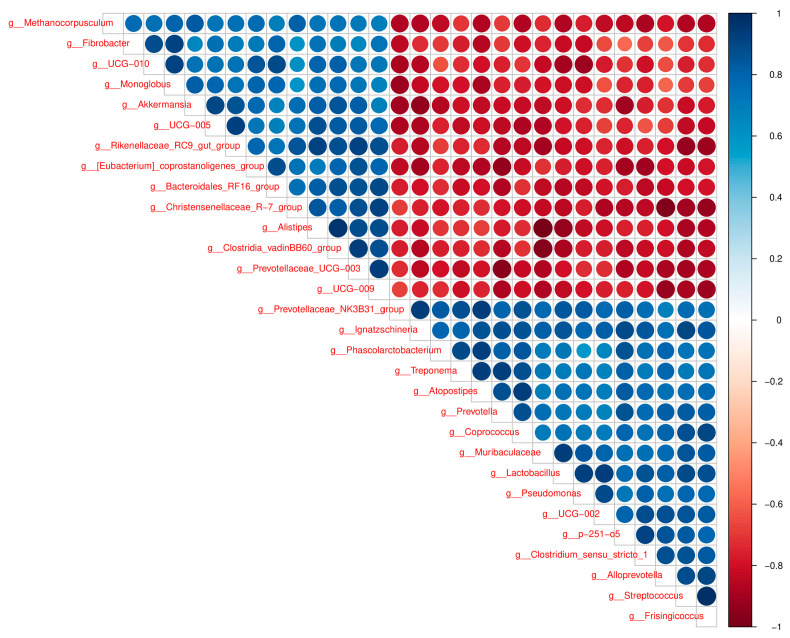
Spearman correlation network of the top 30 differentially abundant bacterial genera between cow and pig dung samples. The strength and direction of correlations reflect the co-occurrence relationships among dominant taxa.

The distinctive abundance of microbial species between cow and pig dung samples was examined using LEfSe, with a standard LDA value > 2 indicating statistical variations between groups. The results showed that 55 taxa had relatively high expression levels in cow dung samples, including f__*Methanocorpusculaceae*, o__*Methanomicrobiales*, c__*Methanomicrobia*, and p__*Halobacterota*, whereas 53 taxa had relatively high expression levels in pig dung samples, including f__*Atopobiaceae*, o__*Coriobacteriales*, c__*Coriobacteriia*, and p__*Actinobacteriota* (Figure 5). According to LDA, which presents the expression levels across taxonomic ranks, including the phylum, class, order, family, and genus, 97 taxa showed relatively high expression levels in cow dung samples, such as o__*Oscillospirales*, c__*Clostridia*, p__*Firmicutes*, f__*UCG-010*, g__*UCG-010*, g__*UCG-005*, f__*Oscillospiraceae*, k__*Archaea*, p__*Halobacterota*, and c__*Methanomicrobia*. In addition, 123 taxa showed relatively high expression levels in pig dung samples, including c_*Gammaproteobacteria*, p__*Proteobacteria*, g__*Pseudomonas*, f__*Pseudomonadaceae*, o__*Pseudomonadales*, f__*Prevotellaceae*, o__*Lactobacillales*, o__*Lachnospirales*, f__*Lachnospiraceae*, and g__*Prevotella* (Figure S4). g__*Methanocorpusculum*, g__*Fibrobacter*, g__*UCG−010*, g__*Monoglobus*, g__*Akkermansia*, and g__*UCG−005* showed strong positive correlations with each other in cow dung samples. g__*Frisingicoccus*, g__*Streptococcus*, g__*Alloprevotella*, g__*Clostridium_sensu_stricto_1*, g__*p−251−o5*, and g__*UCG−002* showed strong positive correlations with each other in pig dung samples (Figure 6).

### 3.6. Microbial Community Composition at Phylum and Genus Levels in Pig and Cow Dung Samples

**Figure 7:**
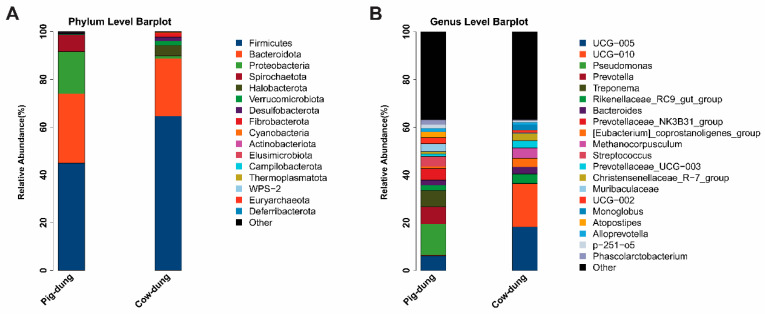
Relative abundances of microbial communities between pig dung and cow dung samples at the phylum (**A**), and genus (**B**) levels. Different microbial taxa at each level are distinguished by various colors.

The final sequencing results identified 106 genera of bacteria in cow dung samples and 144 genera of bacteria in pig dung samples (Tables S4 and S5). The top 20 most abundant bacteria at different taxonomic levels were analyzed. At the phylum level, the most abundant phyla in each group were *Firmicutes* and *Bacteroidetes* in both cow and pig dung samples (Figure 7A). Compared with cow dung samples, the abundance of *Proteobacteria* was markedly reduced and that of *Spirochaetota* increased in pig dung samples. At the genus level, the abundances of *UCG–005* and *UCG*–*010* were clearly reduced in pig dung samples compared with those in cow dung samples, whereas the abundances of *Pseudomonas*, *Prevotella*, and *Treponema* were markedly increased in pig dung samples (Figure 7B). These findings reveal the dominant microbial communities in the two types of dung in rural China.

### 3.7. Analysis of Metabolic Pathways Between Cow and Pig Dung Samples

**Figure 8:**
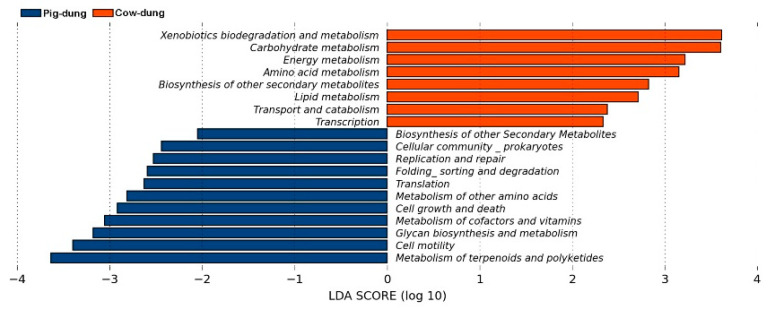
Differential metabolic pathways of bacterial communities between cow and pig dung samples at the KEGG Level 2.

Through metabolic pathway analysis, KEGG results showed that 26 pathways at KEGG-L2 and 136 pathways at KEGG-L3 were identified. LEfSe was used to identify metabolic pathways that significantly impacted sample classification. The default screening criterion was set to LDA > 2. At the KEGG-L2 level, xenobiotic biodegradation and metabolism, carbohydrate metabolism, energy metabolism, and amino acid metabolism were higher in pig than in cow dung samples. The metabolism of terpenoids and polyketides, cell motility, glycan biosynthesis and metabolism, and metabolism of cofactors and vitamins was higher in cow than in pig dung samples (Figure 8). At the KEGG-L3 level, the metabolism of xenobiotics by cytochrome P450, tropane-piperidine, pyridine alkaloid biosynthesis, atrazine degradation, and fatty acid biosynthesis were higher in pig than in cow dung samples. Polyketide sugar unit biosynthesis, bacterial chemotaxis, lipopolysaccharide biosynthesis, and flagellar assembly were higher in cow than in pig dung samples (Figure S5).

### 3.8. Analysis of Specific Microorganisms Between Cow and Pig Dung Samples

**Figure 9:**
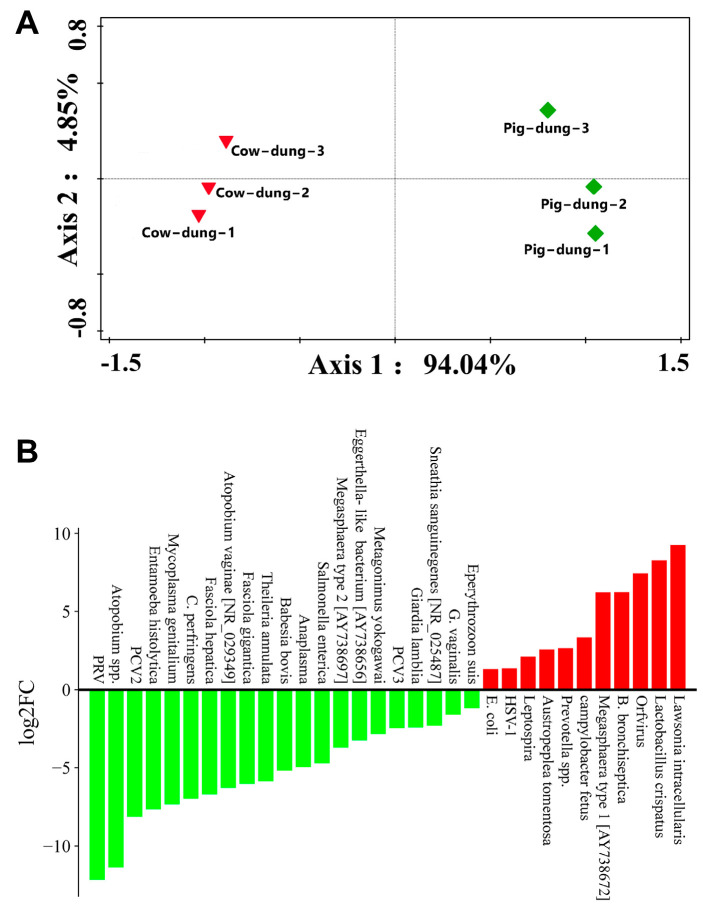
Fluorescent quantitative amplification and differences of specific microorganisms in cow and pig dung samples. (**A**) PCoA of different cow and pig dung samples. (**B**) FC of specific microorganisms between the cow and pig dung samples. Red and green represent up- and downregulated genes, respectively.

**Figure 10:**
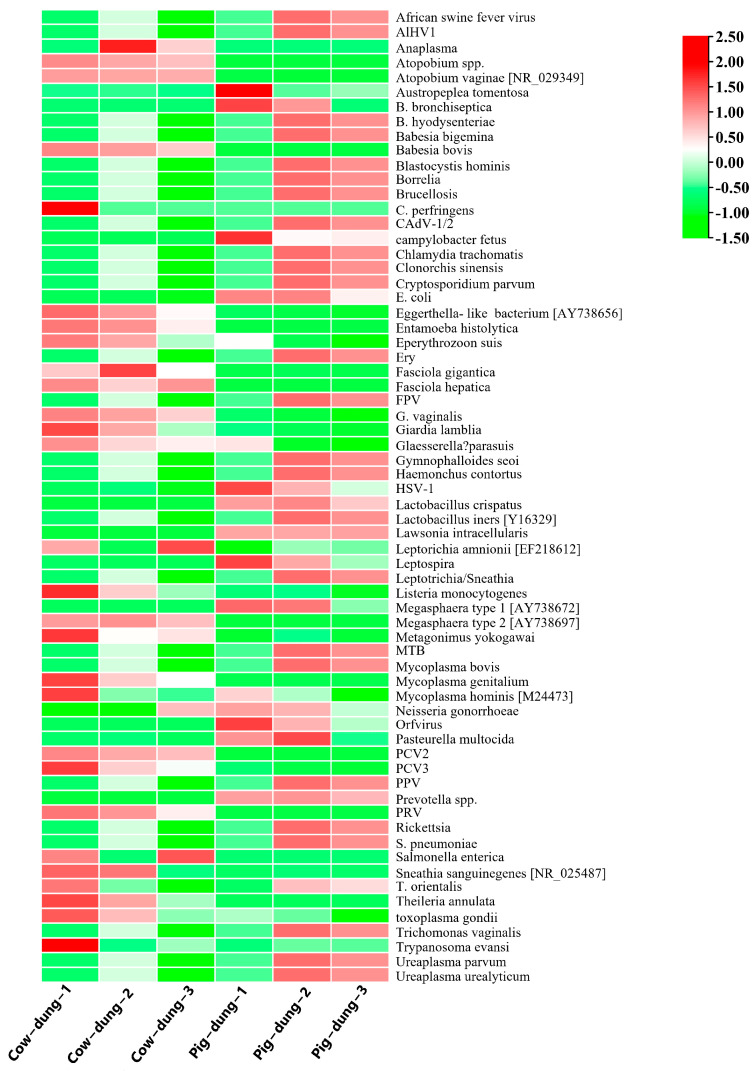
Expression heatmaps of specific microorganisms in different cow and pig dung samples.

The differential abundances of specific microorganisms were investigated. PCoA distinguished cow and pig dung samples, with each group showing distinct clusters (Figure 9A). We developed a method for detecting African pig fever virus using digital PCR but did not detect the nucleic acid sequence of this virus. To further screen for differentially expressed microorganism genes in cow and pig dung samples, we calculated the relative expression abundance based on the log2FC formula. We found that compared with cow dung samples, 21 specific microorganisms including PRV, *Atopobium* spp., porcine circovirus type 2, *Entamoeba histolytica*, *Mycoplasma genitalium*, *Clostridium perfringens*, *Fasciola hepatica*, *Atopobium vaginae* [NR_029349], *Fasciola gigantica*, *Theileria annulata*, *Babesia bovis*, *Anaplasma*, *Salmonella enterica*, *Megasphaera* type 2 [AY738697], *Eggerthella*-like bacterium [AY738656], *Metagonimus yokogawai*, porcine circovirus 3, *Giardia lamblia*, *Sneathia sanguinegenes* [NR_025487], *Gardnerella vaginalis*, and *Eperythrozoon suis* were downregulated in pig dung samples, whereas 11 specific microorganisms including *E. coli*, HSV-1, *Leptospira*, *Austropeplea tomentosa*, *Prevotella* spp., *C. fetus*, *Megasphaera* type 1 [AY738672], *Bordetella bronchiseptica*, *Orfvirus*, *L. crispatus*, and *L. intracellularis* were upregulated (Figure 9B). Because of the differential expression of specific microorganisms between cow and pig dung samples, we analyzed the FC and *p*-value distribution of genes in these microorganisms. Using the criteria of log_2_FC > 1 and *p* < 0.05, we identified 25 differentially expressed specific microorganism genes between cow and pig dung samples, including 16 downregulated and nine upregulated genes. Compared with cow dung samples, 16 specific microorganisms genes including PRV, *Atopobium* spp., porcine circovirus type 2, *E. histolytica*, *M. genitalium*, *F. hepatica*, *A. vaginae* [NR_029349], *F. gigantica*, *T. annulata*, *B. bovis*, *Megasphaera* type 2 [AY738697], *Eggerthella*-like bacterium [AY738656], *M. yokogawai*, porcine circovirus 3, *G. lamblia*, and *G. vaginalis* were downregulated in pig dung samples, whereas nine specific microorganisms genes, including *E. coli*, HSV-1, *Leptospira*, *Prevotella* spp., *C. fetus*, *Megasphaera* type 1 [AY738672], *Orfvirus*, *L. crispatus*, and *L. intracellularis* were upregulated (Figure S6). Genera enriched in pig dung, including *Escherichia* and *Leptospira*, are known ARG hosts, suggesting that microbial composition contributes to the higher ARG abundance observed in pig dung. We analyzed 66 specific microorganisms in each sample. The results showed the differential microbial abundance profiles (Figure 10).

## 4. Discussion

This study analyzed the microbial communities and ARG profiles of cow and pig dung from rural China. The results showed higher ARG levels in pig dung, which was also enriched in conditional pathogens such as *E. coli* and *Leptospira*. Cow dung contained more bacteria related to ruminant digestion, such as *UCG-005* and *Methanocorpusculum*. Metabolic pathway analysis revealed stronger xenobiotic degradation and amino acid metabolism in pig dung, whereas pathways in cow dung were related to energy metabolism and bacterial chemotaxis. In addition, pig dung contained high levels of mobile genetic elements, suggesting a high risk of ARG transfer. These findings may contribute to the management of antibiotic resistance and improve the health of livestock in rural China. However, as all samples were collected in a single region and season, potential variation caused by regional or seasonal factors cannot be fully excluded. Future work should include multiple seasons and locations to better capture environmental diversity.

Our findings revealed striking differences in the ARG abundance between pig and cow dung, with significantly higher levels of ARGs in pig dung (56 upregulated genes) than in cow dung (8 upregulated genes), which are clinically relevant and associated with human and animal pathogens. Previous studies detected *E. coli* producing extended-spectrum β-lactases in animal and environmental samples from four pig farms in Italy [25]. Lebanese pig farms showed a high prevalence of Gram-negative Bacilli producing extended-spectrum β-lactase and the emergence of the *mcr-1* colistin resistance gene [26]. Notably, pig dung exhibited enrichment of clinically relevant ARGs, such as β-lactamase genes (*blaCMY*, *blaCTX-M*) and macrolide resistance genes (*ermB*, *ermF*), spanning nine resistance groups, including aminoglycosides and MLSB (macrolide-lincosamide-streptogramin B). These results are consistent with those of previous studies linking monogastric animal diets (often high in antibiotics and nutrients) with elevated ARG expression [27,28,29]. The low ARG diversity in cow dung may reflect the protective role of ruminant microbiomes in reducing antibiotic selective pressure or the dilution effect of fiber-rich diets [30]. The ARG *intI1* was shown to be prevalent in pig wastewater treatment plants in a previous study [31].

The high abundance of mobile genetic elements (*intI1* and *IS613*) in pig dung highlights the critical risk of horizontal ARG transfer, a process facilitated by Proteobacteria and opportunistic pathogens (*Pseudomonas* and *Streptococcus*) that dominate pig microbial communities [32,33]. These genes can be easily transferred to pathogenic bacteria, potentially leading to treatment failure in human infections and jeopardizing food safety [34]. In contrast, our study showed that cow dung was enriched in methanogens (*Methanocorpusculum*) and *fibrolytic* bacteria (*Fibrobacter*, *UCG-005*), which are integral to ruminant digestion but less associated with ARG dissemination [35,36]. These results highlight pig dung as a hotspot for ARG proliferation and horizontal transfer, posing greater risks during agricultural reuse. Coexistence of genera such as g__*UCG-005*, g__*UCG-010*, g__*Rikenellaceae*_RC9_gut_group, and g__[*Eubacterium*]_*coprostanoligenes*_group was observed in previous studies [37,38,39]. We found that g__*Prevotellaceae*_*UCG-003*, g__*Bacteroides*, and others were significantly expressed, further expanding the microbial composition of cow dung under feeding conditions. g__*Pseudomonas*, g__*Prevotella*, and g__*Treponema* coexist in the intestines of pigs [40,41,42]. Previous studies revealed the coexistence of g__*UCG-005*, g__*Prevotellaceae*_NK3B31_group, and g__*Streptococci* in pigs. Our study expanded the composition categories of intestinal bacteria in monogastric and compound gastric animals by comparing their dung microbiota. Revealing distinct ecological patterns in cow and pig dung.

Cow dung is characterized by Archaea and *fibrolytic* bacteria, reflecting adaptations to cellulose digestion and methane production, which are critical for ruminant energy metabolism [43]. In contrast, pig dung contains a higher abundance of Proteobacteria and opportunistic pathogens, consistent with their roles in nutrient absorption and susceptibility to bacterial infections in monogastric systems [44,45]. Metabolic pathway analysis further highlighted functional divergence. Pig dung exhibited stronger activities in xenobiotic degradation and amino acid metabolism, potentially linked to antibiotic metabolism and pathogen survival, whereas cow dung prioritized energy metabolism and bacterial chemotaxis, supporting fiber digestion and microbial motility. These functional differences may drive ARG persistence in pig dung, as xenobiotic degradation pathways often co-occur with antibiotic resistance mechanisms [46]. Although diverse ARGs were identified, their functional activity and transfer potential were not verified in this study. Future work should employ culture-based validation, such as conjugation or transformation experiments, to evaluate the real-world risk of ARG dissemination from livestock dung.

Some microorganisms with high expression levels in cow dung were classified into three major categories: viruses, bacteria, and parasites. For example, porcine circovirus, porcine circovirus 2, and porcine circovirus 3 are viruses; bacteria include *Atopobium* spp. and *A. vaginae*. *Mycoplasma genitalium*, *Megasphaera* type 2, and *Eggerthella*-like bacteria are bacteria [47,48,49,50,51,52]. In addition, *E. histolytica*, *T. annulata*, *B. bovis*, *G. lamblia*, *F. hepatica*, *F. gigantica*, and *M. yokogawai* were detected as parasites [53,54,55,56,57,58], underscoring their role in transmitting protozoan diseases, albeit with a lower ARG burden in cow dung. Our research also revealed the presence of microorganisms such as HSV-1, *Prevotella* spp., *C. fetus*, *Megasphaera* type 1, Orfvirus, and *L. crispatus*, which were previously less frequently detected in pigs [59,60,61,62]. We found that *Eggerthella*-like bacteria are expressed in both cow and pig dung, with particularly high expression levels in cow dung. Additionally, PRV had the highest content in cow dung, and *L. intracellularis* had the highest expression level in pig dung. These results provide important information for further research on animal and human public health. Although African pig fever virus was not detected, co-enrichment of pathogens and ARGs in pig dung created a “dual-risk” composite contaminant, increasing the likelihood of zoonotic disease transmission and spread of antibiotic resistance via dung-amended soils or water bodies. The presence of MGEs in the pig microbiota further amplifies these risks by enabling ARG dissemination across bacterial taxa, including human pathogens. This study presents an integrated analysis of ARGs, microbiomes, and pathogens in rural Chinese livestock dung, leveraging high-throughput qPCR and 16S sequencing to capture multilevel biological signals [63].

By focusing on small-scale farms, a common but understudied system in developing countries, we provide context-specific data on how non-standardized practices drive ARG and pathogen enrichment [64,65]. These findings are critical for tailoring dung management strategies in rural Asia, where composting and biogas engineering are widespread but often lack ARG mitigation protocols. Furthermore, we explored how livestock management practices influence these profiles, thereby contributing valuable insights into the ecological implications of antibiotic use in agriculture [66,67]. This foundational knowledge is crucial for developing effective strategies to mitigate the spread of antibiotic resistance from livestock to humans, ultimately enhancing public health and food safety [68,69]. Collectively, our results may lead to innovative approaches in livestock management and antibiotic stewardship, ultimately contributing to reductions in public health risks associated with antibiotic-resistant bacteria originating from agricultural sources [70,71,72]. Although the results provide robust insights into dung microbiomes and ARGs, we did not address the spatial variability in dung composition or the long-term environmental fate of ARGs after dung application. Further studies involving metagenomics are needed to link ARGs to specific microbial hosts and to assess ARG persistence in agricultural ecosystems. In addition, evaluating the efficacy of composting or anaerobic digestion in reducing ARGs and pathogens in pig dung can inform sustainable waste management practices. The integration of ARG profiles and microbial community data revealed that the dominance of potential ARG-carrying genera, such as *Escherichia* and *Leptospira*, in pig dung may underlie its higher ARG abundance and indicate a greater risk of resistance dissemination to the environment and humans.

This study has several limitations that should be acknowledged. First, the sample sizes used for both qPCR and 16S rDNA sequencing were relatively small, and dung samples were collected from only two smallholder farms. These factors restrict the statistical power and generalizability of the findings. The two farms were located in different rural areas of Kaifeng, and regional differences in livestock management or environmental conditions may have contributed to variability in microbial community structure and antibiotic resistance profiles. Moreover, both farms operated under non-standardized management, where diverse feed sources, lack of sanitation, and undocumented antibiotic use may have influenced dung quality and microbiota composition. In addition, the study primarily relied on sequencing-based analyses, with limited experimental validation to support the inferred functional characteristics of microbial communities and ARGs.

Despite these constraints, this work provides foundational insight into microbial and ARG patterns in small-scale, non-standardized livestock systems—an understudied yet widely prevalent farming model in rural China. To strengthen and expand these findings, future studies should incorporate larger sample sizes, include farms representing different management modes (standardized, intensive, and semi-intensive), and systematically examine spatial and seasonal variability. Integrating metagenomic, functional, and experimental approaches, as well as evaluating the long-term effects of manure application on soil health and microbial dynamics, will be essential for establishing a more comprehensive framework for assessing microbial and antibiotic resistance risks associated with livestock manure.

## 5. Conclusions

This study reveals distinct microbial and antibiotic resistance profiles in pig and cow dung collected from different small-scale rural farms, demonstrating that pig dung carries a higher abundance and diversity of ARGs as well as a broader range of potential pathogens. By integrating microbial community, ARG, and metabolic pathway analyses, we provide a comprehensive framework for characterizing dung-derived antibiotic resistance under non-standardized smallholder farming conditions. The elevated levels of ARGs and mobile genetic elements in pig dung suggest that pig manure may serve as a particularly important reservoir and potential source of resistance dissemination during agricultural reuse. However, we acknowledge that the limited sample size and the inclusion of only two smallholder farms restrict the generalizability of these findings. Despite these constraints, the results offer valuable baseline information for understanding microbial and ARG accumulation patterns in under-documented smallholder livestock systems. These findings underscore the importance of adopting “One Health”–oriented strategies—such as prudent antibiotic use, effective composting, and biogas treatment—to limit ARG spread and protect environmental and public health. Future research should expand sampling across more farms, management modes, geographic regions, and seasons, and verify ARG functionality through metagenomic and experimental approaches. Additionally, evaluating the performance of diverse dung treatment technologies will be essential for developing sustainable livestock waste management policies that minimize antibiotic resistance risks in agricultural ecosystems.

## Data Availability

All data generated or analyzed during this study are included in the article.

## Supplementary Materials

The following supporting information can be downloaded at: https://www.mdpi.com/article/10.3390/biology14111623/s1, Figure S1. Volcano plot of differentially expressed antibiotic resistance genes (ARGs) between cow and pig dung samples. Red and green dots represent genes upregulated and downregulated, respectively, in pig dung samples. Figure S2. Analysis of amplicon sequence variants (ASVs) in cow and pig dung samples. (A) Principal Component Analysis (PCA) based on ASV abundance. Percentages on the axes indicate the contribution of each principal component to total variance. Different colors represent different sample groups, and larger distances indicate greater differences in microbial community composition. (B) Venn diagram showing the total number of ASVs shared between groups and those unique to each group. ASV, amplicon sequence variant; PCA, principal component analysis. Figure S3. Beta diversity analysis of bacterial communities in cow and pig dung samples. (A) Principal Coordinates Analysis (PCoA) plot. (B) Non-metric Multidimensional Scaling (NMDS) plot. (C) Analysis of similarities (ANOSIM) plot. (D) Unweighted UniFrac heatmap. PCoA, principal coordinates analysis; NMDS, non-metric multidimensional scaling. Figure S4. Linear Discriminant Analysis (LDA) scores of bacterial taxa at the phylum, class, order, family, and genus levels (LDA threshold = 2). Figure S5. Differential bacterial metabolic pathways between cow and pig dung samples at the KEGG Level 2 classification. Figure S6. Volcano plot of differentially abundant microorganisms between cow and pig dung samples. FC, fold change; PCoA, principal coordinates analysis. Table S1. List of antibiotic resistance genes (ARGs) and their specific primer sequences used for detection. Table S2. List of microbial species and their corresponding primer sequences. Table S3. Summary of sequencing data for bacterial communities in cow and pig dung samples. Table S4. Bacterial genera (*n* = 106) identified in cow dung samples. Table S5. Bacterial genera (*n* = 144) identified in pig dung samples.
