# Supplementary figures and images for: Comparative Profiling of Antibiotic Resistance Genes and Microbial Communities in Pig and Cow Dung from Rural China: Insights into Environmental Dissemination and Public Health Risks
Source: Biology (Basel). 2025 Nov 19;14(11):1623. doi: 10.3390/biology14111623 (PMC12650751; doi:10.3390/biology14111623)

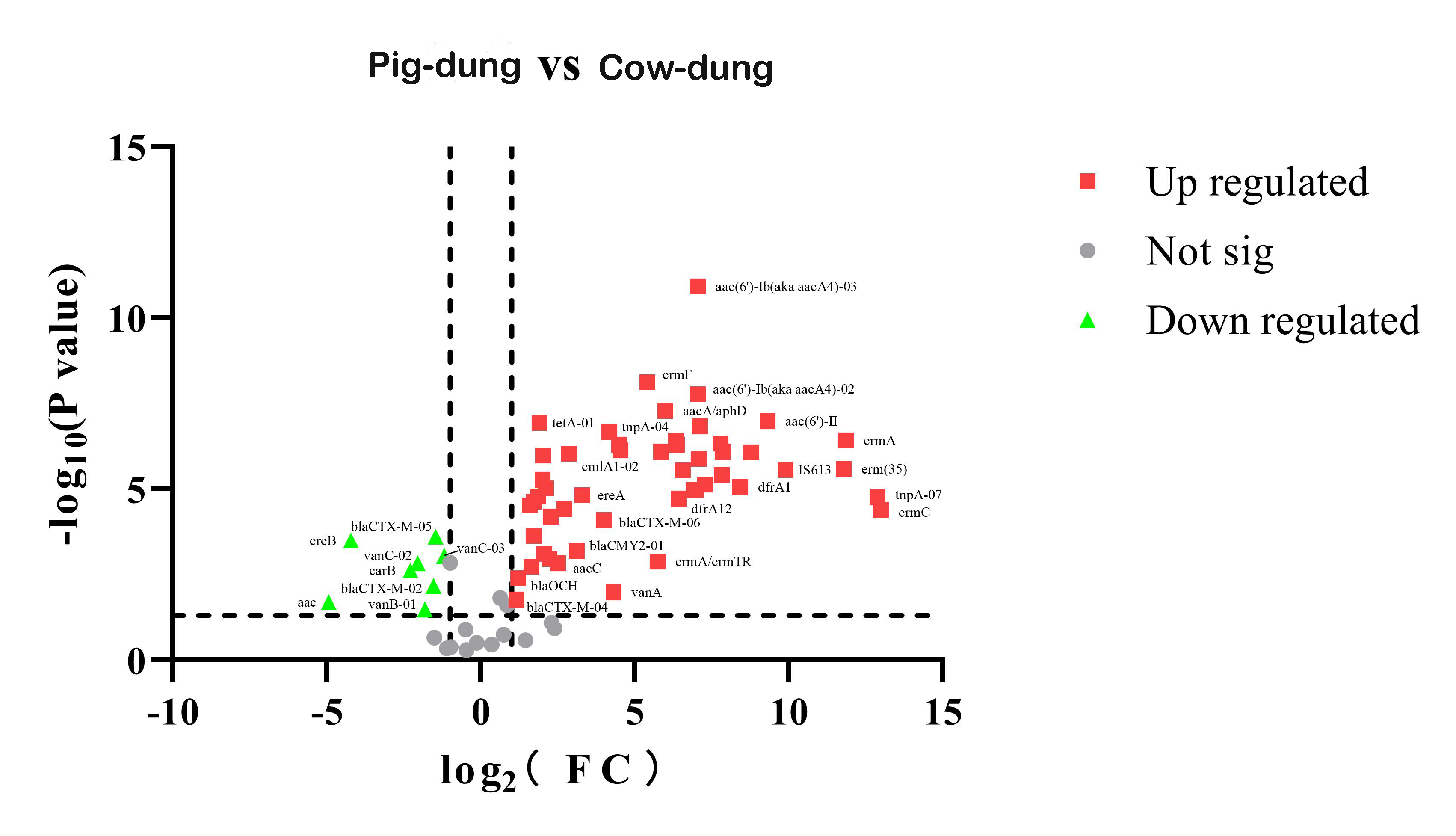

Supplement: Supplementary file 1 [file biology-14-01623-s001.zip › Figure S1.jpg]

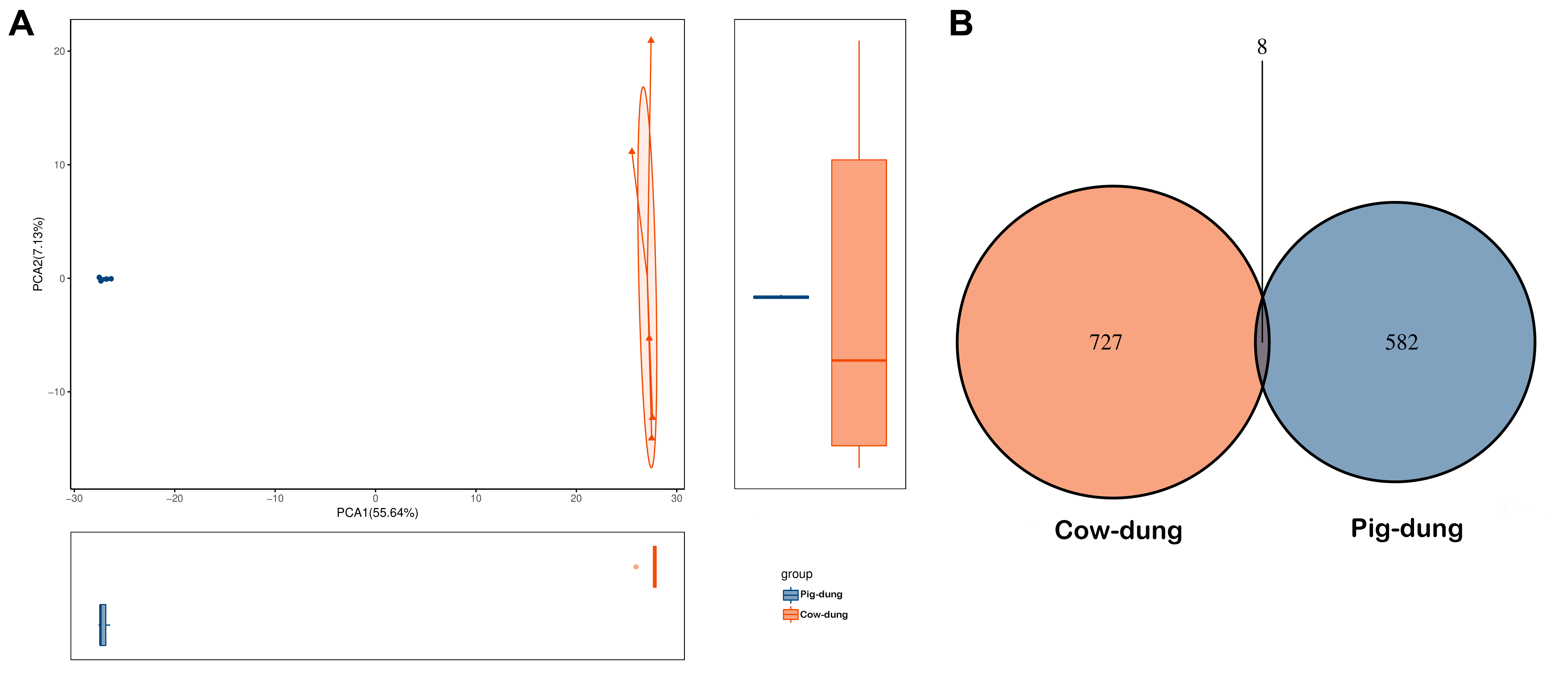

Supplement: Supplementary file 1 [file biology-14-01623-s001.zip › Figure S2.tif]

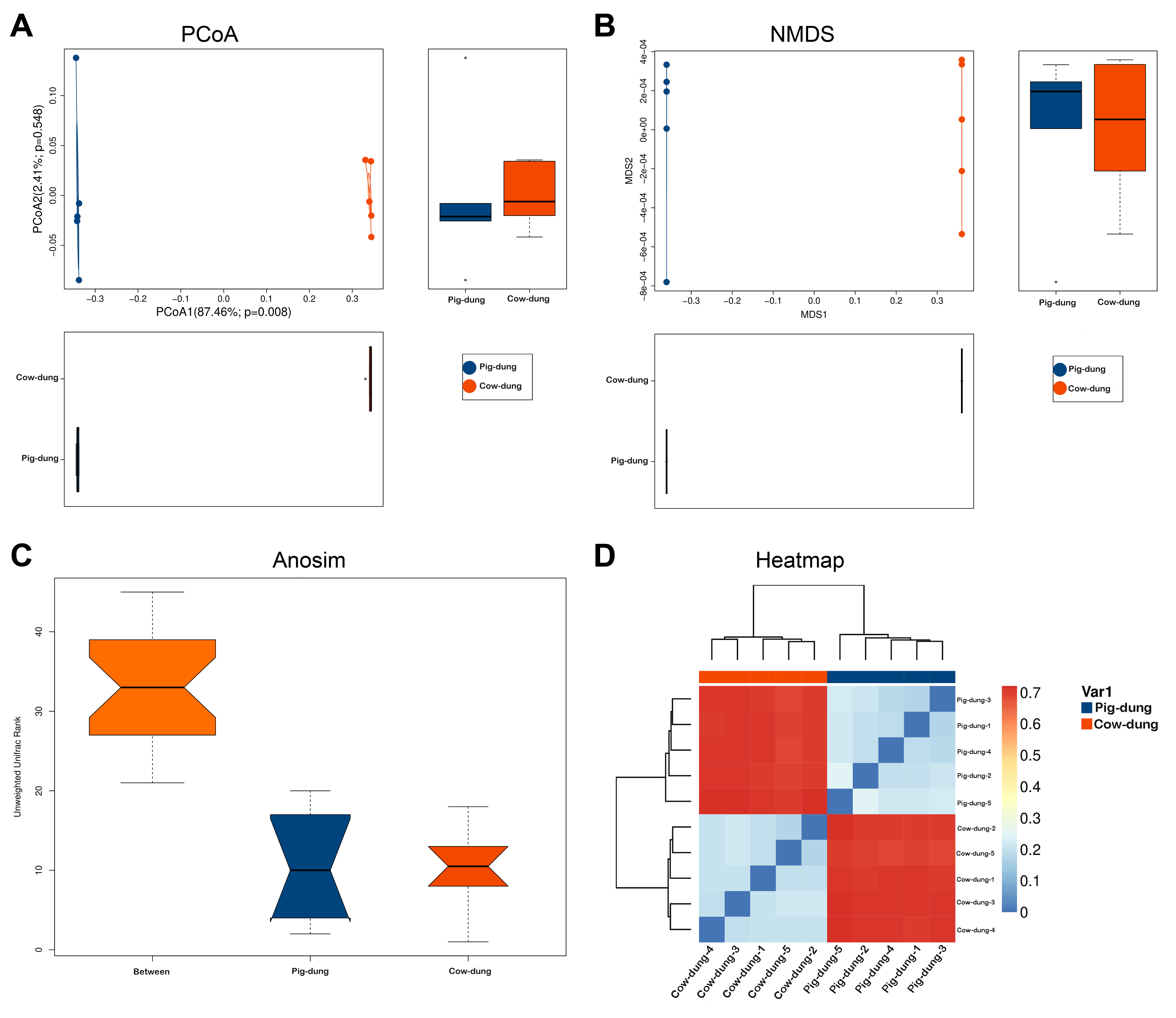

Supplement: Supplementary file 1 [file biology-14-01623-s001.zip › Figure S3.tif]

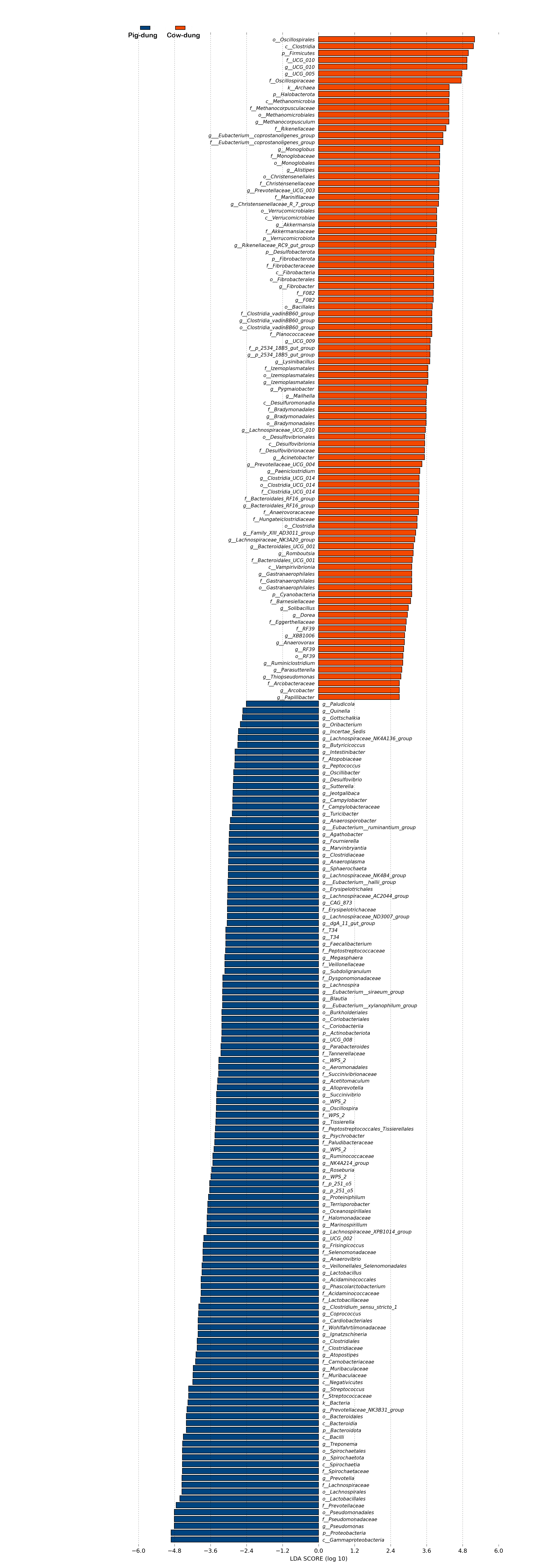

Supplement: Supplementary file 1 [file biology-14-01623-s001.zip › Figure S4.jpg]

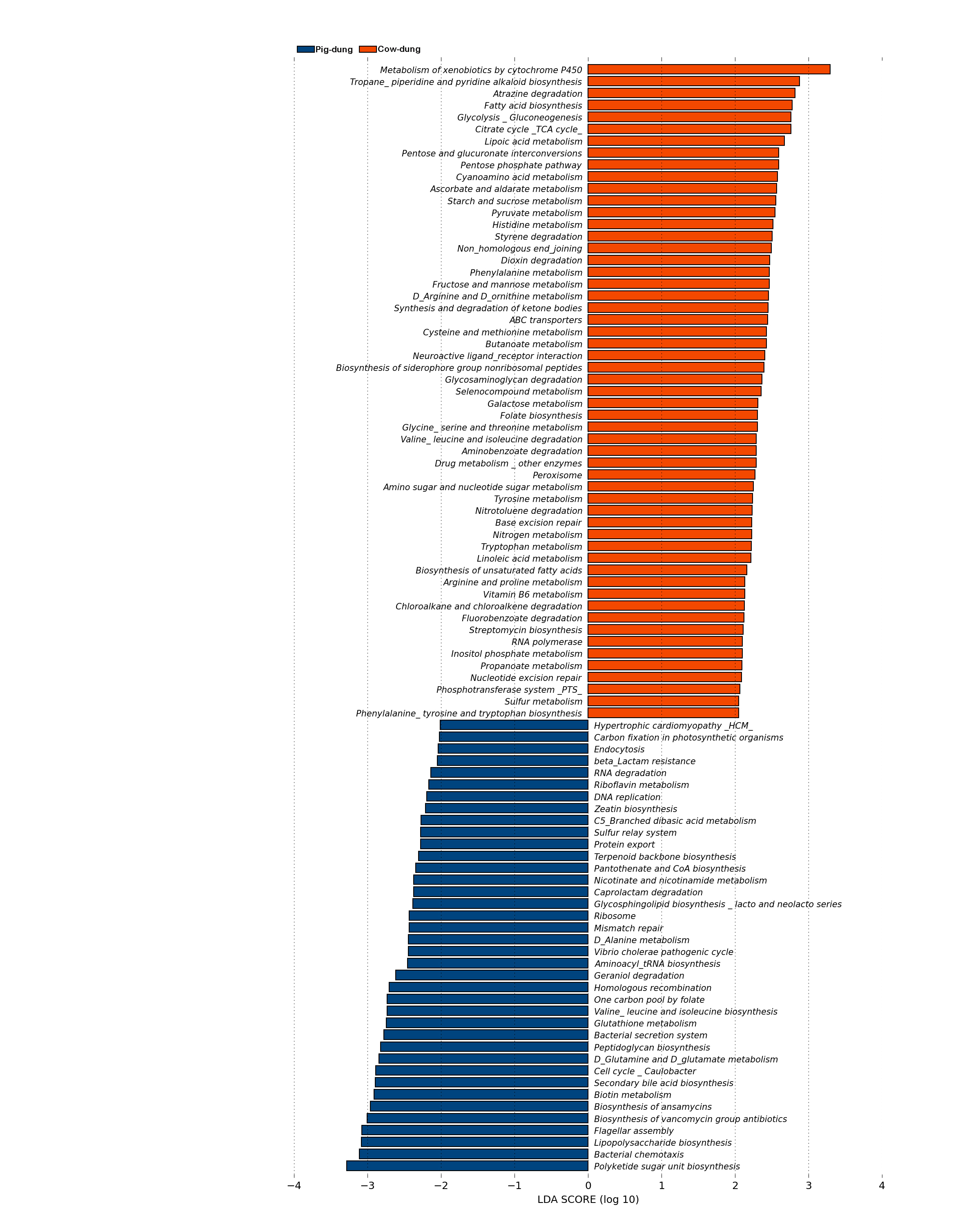

Supplement: Supplementary file 1 [file biology-14-01623-s001.zip › Figure S5.jpg]

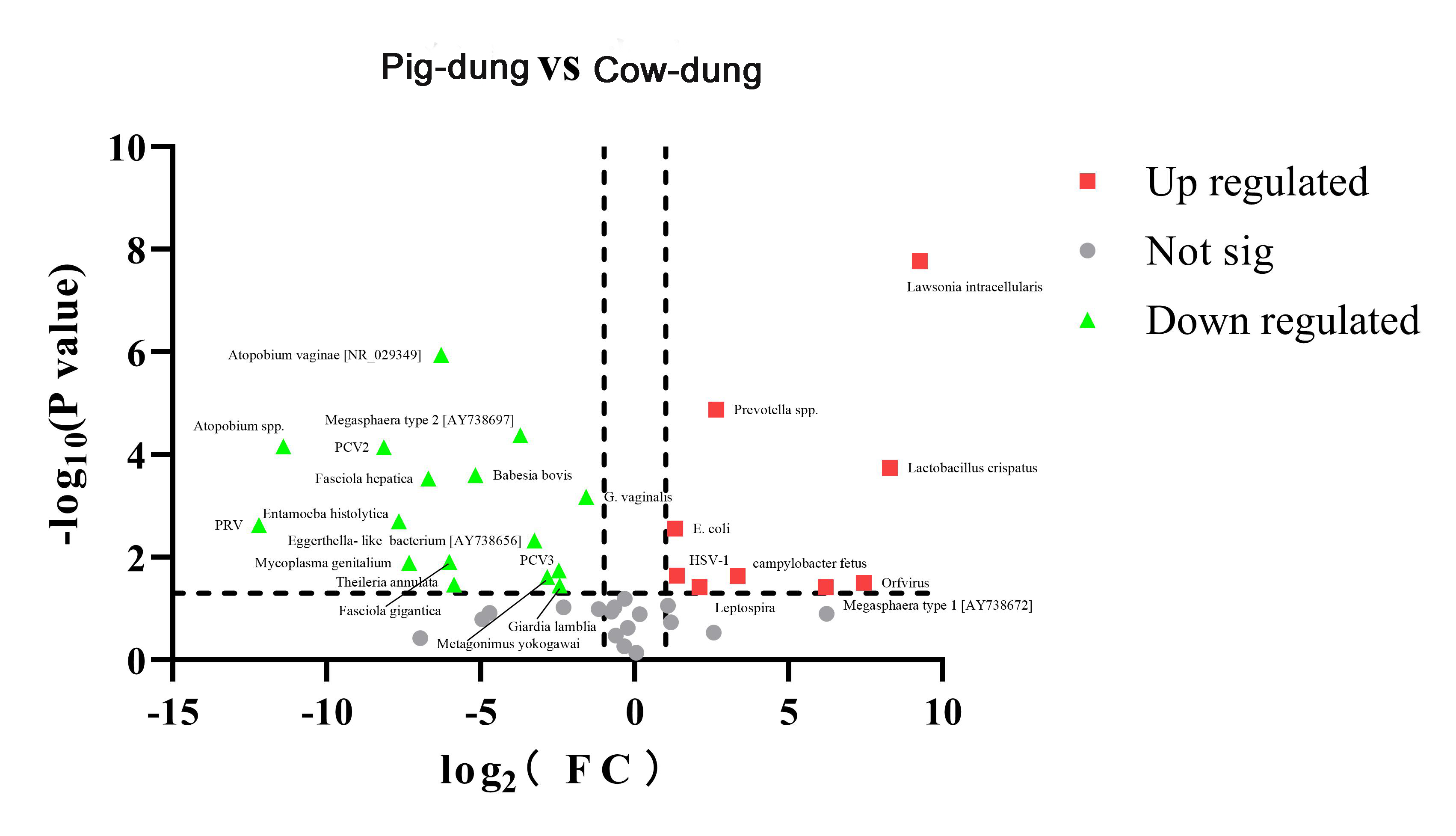

Supplement: Supplementary file 1 [file biology-14-01623-s001.zip › Figure S6.jpg]
